# Vitamin E supplementation and pneumonia risk in males who initiated smoking at an early age: effect modification by body weight and dietary vitamin C

**DOI:** 10.1186/1475-2891-7-33

**Published:** 2008-11-19

**Authors:** Harri Hemilä, Jaakko Kaprio

**Affiliations:** 1 Department of Public Health, POB 41, University of Helsinki, Helsinki, FIN-00014, Finland

## Abstract

**Background:**

We had found a 14% higher incidence of pneumonia with vitamin E supplementation in a subgroup of the Alpha-Tocopherol Beta-Carotene Cancer Prevention (ATBC) Study cohort: participants who had initiated smoking by the age of 20 years. In this study, we explored the modification of vitamin E effect by body weight, because the same dose could lead to a greater effect in participants with low body weight.

**Methods:**

The ATBC Study recruited males aged 50–69 years who smoked at least 5 cigarettes per day at the baseline; it was conducted in southwestern Finland in 1985–1993. The current study was restricted to 21,657 ATBC Study participants who initiated smoking by the age of 20 years; the median follow-up time was 6.0 years. The hospital-diagnosed pneumonia cases were retrieved from the national hospital discharge register (701 cases).

**Results:**

Vitamin E supplementation had no effect on the risk of pneumonia in participants with body weight in a range from 70 to 89 kg (n = 12,495), risk ratio (RR) = 0.99 (95% CI: 0.81 to 1.22). Vitamin E increased the risk of pneumonia in participants with body weight less than 60 kg (n = 1054), RR = 1.61 (1.03 to 2.53), and in participants with body weight over 100 kg (n = 1328), RR = 2.34 (1.07 to 5.08). The harm of vitamin E supplementation was restricted to participants with dietary vitamin C intake above the median.

**Conclusion:**

Vitamin E supplementation may cause harmful effects on health in certain groups of male smokers. The dose of vitamin E used in the ATBC Study, 50 mg/day, is substantially smaller than conventional vitamin E doses that are considered safe. Our findings should increase caution towards taking vitamin E supplements.

**Trial registration:**

ClinicalTrials.gov NCT00342992.

## Background

According to a recent survey, about half of 60 years and older US adults took supplements containing vitamin E, and half of them took high doses, ≥ 400 IU/day [1]. Such a common habit makes the health effects of this practice an important public health issue and raises the question of whether taking vitamin E supplements is beneficial or not.

The rationale behind vitamin E supplementation is based on the protection against oxidative stress, which has a role in diverse aging processes [2,3]. However, in randomized trials vitamin E supplementation has not reduced mortality implying that it has no substantial effect on the processes leading to chronic diseases [4-6].

Specifically, vitamin E supplementation has been advocated for improving the immune system [7,8]. A number of animal studies found that vitamin E reduced the incidence and severity of various viral and bacterial infections [9]. On the other hand, vitamin E shortened the survival of mice with malaria [10,11] and increased the multiplication of *Leishmania *parasites in hamsters [12]. Two studies in human subjects found that large doses of vitamin E depressed bactericidal activity of phagocytes [13,14]. Thus, vitamin E supplementation can also be harmful to the immune system.

Vitamin E is a major lipid soluble anti-oxidant whereas vitamin C is a major water-soluble antioxidant. They interact in vitro and in vivo [9,15-17]. Smoking increases the plasma α-tocopherol disappearance rate, which is normalized by vitamin C supplementation [17]. Thus, smoking seems to modify the interaction between these two antioxidants. Therefore, vitamin C is particularly important when examining the effects of vitamin E supplementation on smokers.

Previously we analyzed the Alpha-Tocopherol Beta-Carotene Cancer Prevention (ATBC) Study cohort and found significant heterogeneity in the effect of vitamin E supplementation on the incidence of the common cold, pneumonia and tuberculosis [18-20]. Vitamin E reduced pneumonia risk by 35% in 7,469 participants who initiated smoking at 21 years or later, but in those who initiated smoking earlier the vitamin elevated pneumonia risk nonsignificantly by 14% [18]. Furthermore, the subgroup of participants who initiated smoking late was so large (n = 7,469) that we carried out second-level subgroup analyses and found significant heterogeneity in the effect of vitamin E supplementation by the level of smoking [18]. The group of participants who initiated smoking at early age is even larger (n = 21,657) and therefore further subgroup analysis is justified to examine whether there is heterogeneity in vitamin E effect also in this subgroup.

The ATBC Study was a randomised, double-blind, placebo-controlled trial which examined the effects of vitamin E and β-carotene on lung cancer in male smokers using a 2 × 2 factorial design [21,22]. A single dose of vitamin E, 50 mg/day (50 IU/day), was used and therefore the dose-response of vitamin E supplementation cannot be examined directly. Nevertheless, the dose-response can be explored indirectly by examining the variation of vitamin E effect by body weight. Thus, we anticipated a greater effect on those who had the lowest body weight.

The primary objective of this study was to examine whether body weight modifies the increased pneumonia risk by vitamin E supplementation in the 21,657 ATBC Study participants who had initiated smoking at an early age [18]. As a secondary objective we explored whether dietary vitamin C intake and other major baseline variables might modify the effect of vitamin E supplementation.

## Methods

### Participants

The design and methods of the ATBC Study examining the effects of vitamin E (*dl*-α-tocopheryl acetate, AT, 50 mg/day) and β-carotene (BC, 20 mg/day) on the incidence of lung cancer and other cancers have been described in detail [18,21,22]. The ATBC Study is registered at ClinicalTrials.gov under the identifier NCT00342992.

In brief, to be eligible, male participants aged 50–69 years had to smoke ≥ 5 cigarettes per day at entry, and those enrolled in the trial (N = 29,133) were randomised to one of four intervention arms and administered placebo, AT, BC, or AT+BC, using a 2 × 2 factorial design. Compared with the baseline levels, supplementation increased the serum level of α-tocopherol by 50% [22]. The intervention continued for 5 to 8 years until April 1993. The trial was approved by the institutional review boards and all participants gave written informed consent. We restricted the present analysis to participants who started to smoke at ≤ 20 years (N = 21,657), with 10,784 participants who were administered vitamin E (AT and AT+BC) and 10,873 participants who were not administered vitamin E (placebo and BC).

### Baseline characteristics

Before randomisation at baseline, the men completed questionnaires on medical and smoking histories and general background characteristics, and height and weight were measured. A detailed dietary history questionnaire was completed that provided data regarding vitamin C, vitamin E, fruit, vegetable, and berry consumption [23]. Body-mass-index (BMI) was calculated as weight/height^2 ^[kg/m^2^]. Dietary data were not available for 1463 of the 21,657 participants. Weight was not available for 12, height for 11 and BMI for 14 participants.

### Outcome and follow-up time

The events for this study, the first hospital-treated pneumonia after randomization, were ascertained from the national Hospital Discharge Register using the unique personal identification number for linkage (see details in Ref. [18]). Pneumonia cases recorded in the Hospital Discharge Register reflect clinically more severe cases of greater health and economic significance, whereas less severe cases of pneumonia treated as outpatients are not recorded in the Register. Because almost all of the ATBC Study participants lived at home, the pneumonia cases ascertained represent community-acquired pneumonia; medical records were not reviewed to rule out the few nosocomial infections.

### Statistical methods

Follow-up time for each participant began from the day of randomization, and continued until the date of first hospital discharge for pneumonia, death, or the end of the trial, April 30, 1993, whichever came first. In cases where both pneumonia and lung cancer were present in the same discharge record (n = 57 cases), follow-up was censored at the date of hospital discharge, but the participant was not classified as a case of pneumonia, as previously [18].

The median follow-up time of the participants in the present analysis was 6.0 years, and there was a total of 124,612 person-years of observation. Use of the Hospital Discharge Register allowed obtaining information on pneumonia in study participants irrespective of whether they continued in or had dropped out from the trial.

We estimated the effect of vitamin E supplementation on pneumonia incidence through proportional hazards regression models. We calculated the risk ratio (RR) and the 95% confidence interval (CI) of the RR using the PROC PHREG program of the SAS package of programs (release 8.2, SAS Institute, Inc., Cary, North Carolina). The 2 × 2 factorial design of the trial permitted assessment of the effect of vitamin E independent of β-carotene after confirming no statistical interaction between the agents. Thus, we compared the trial participants administered vitamin E (AT and AT+BC) with those not receiving vitamin E (the no-vitamin E group; placebo and BC). We did not analyze the effects of β-carotene in this study. As to supplementation, we carried out the analyses following the intention-to-treat principle. When appropriate, we adjusted the statistical models for age, baseline smoking, intake of coffee and BMI as continuous variables, and for the intake of alcohol categorized to 0, > 0–29, 30–59, and ≥ 60 g/day, and being employed categorized to yes/no [18].

To test the statistical significance of interaction between vitamin E supplementation and potential modifying factors, we first added the supplementation and the modifying factor to the regression model. The statistical significance of the interaction was thereafter calculated from the change in -2 × log(likelihood) when the interaction term for vitamin E supplementation and the modifying factor was added to the model. In our subgroup analyses to assess effect modification, we split at the median the BMI, height, dietary vitamin E and C levels, and the residual of fruit, vegetables, and berries (see below). Dietary vitamin C was also used as a continuous variable since interaction with a continuous variable refutes the possibility that dichotomizing a continuous variable may cause a spurious interaction; to decrease the weight of distant points, the logarithm of dietary vitamin C intake was included in the statistical model.

The major vitamin C sources in the diet of the study participants were fruit, vegetables and berries; on average 58% of dietary vitamin C originated from these foods. The total intake of fruit, vegetables and berries (FRUVEBE) was strongly correlated with the calculated vitamin C intake (r = 0.88). Thus, it is possible that an association with dietary vitamin C is a statistical artefact reflecting other substances of these foods or the life style related to eating these foods. To examine the possible role of dietary compounds other than vitamin C in these foods, we calculated the residual of FRUVEBE intake (FBUVEBE-RES) using linear regression to model FRUVEBE as a function of dietary vitamin C, as previously [24]:

FBUVEBE-RES [g/day] = FRUVEBE [g/day] - 2.47 × vitamin C [mg/day] + 36.3 [g/day]

As designed, FRUVEBE-RES had no correlation with dietary vitamin C. We assume that any other putative compounds that might interact with vitamin E supplementation have no perfect linear correlation with vitamin C and therefore variation in the other substances remains as variation in FRUVEBE-RES. High FRUVEBE-RES (over median) indicates that the participant with a given vitamin C level consumes more than the average amount of fruit, vegetables and berries, whereas low FRUVEBE-RES (below median) indicates less than the average intake of these food classes.

Nelson-Aalen cumulative hazard functions were constructed using STATA sts program (Release 9, Stata Corp, College Station, TX). Two-tailed P-values were used.

## Results

Table 1 shows the distribution of the major characteristics of the ATBC Study participants who initiated smoking by the age of 20 years. During 124,612 person-years of follow-up, there were 701 new cases of hospital-treated pneumonia, representing a mean incidence rate of 5.6 cases per 1000 person-years.

**Table 1:** Baseline characteristics of ATBC Study participants initiating smoking at ≤ 20 years

Characteristic	Number of participants
All participants	21657
Age (years)	
50–54	7778
55–59	6908
60–64	5058
65–69	1913
Cigarettes per day at baseline	
5–19	7295
20–29	10259
≥ 30	4103
BMI (kg/m^2^) *	
≤ 19	700
20–24	7781
25–29	9902
≥ 30	3260
Education (years at school)	
≤ 6	17253
7–9	2956
≥ 10	1448
Residential neighborhood during the last 20 years *	
City (> 50,000 inhab.)	9305
Town	4667
Village	3160
Countryside	4519

* BMI was missing for 14 and residential neighborhood for 6 participants.

Overall, the risk of pneumonia was nonsignificantly 14% higher in the vitamin E-supplemented participants compared with the no-vitamin E participants (Table 2). Vitamin E had no effect, RR = 0.99, on those with body weight in the middle range from 70 to 89 kg, which covers the great majority of this cohort. In participants with the lowest and highest body weight, vitamin E supplementation significantly increased the risk of pneumonia (Table 2). In the groups next to the extremes, there was a nonsignificant elevation of pneumonia risk by vitamin E supplementation suggesting a trend. Our further explorative subgroup analyses were focused on the lowest and highest body-weight subgroups (Tables 3 and 4). Restriction to smaller subgroups may lead to unbalance in potential confounders, and therefore we adjusted regression models in Tables 3 and 4 with potential confounders. These tables are restricted to participants for whom the confounders were available.

**Table 2:** Relative risk of hospital-treated pneumonia by vitamin E supplementation in participants initiating smoking at ≤ 20 years, ATBC Study 1985–1993

		Intervention				Effect of vitamin E
		Vitamin E		No vitamin E		
Subgroup	No. of participants	No. of cases	Rate*	No. of cases	Rate*	RR (95% CI)*
All	21657	370	6.0	331	5.3	1.14 (0.98–1.32)
Weight (kg) †						
36–59	1054	47	16.7	32	10.4	1.61 (1.03–2.53)
60–69	4115	79	6.7	68	5.7	1.17 (0.84–1.62)
70–89	12495	182	5.1	187	5.1	0.99 (0.81–1.22)
90–99	2653	39	5.2	34	4.5	1.17 (0.74–1.86)
100–154	1328	22	5.7	9	2.5	2.34 (1.07–5.08)
β-Carotene						
No	10842	204	6.6	169	5.4	1.22 (0.99–1.50)
Yes	10815	166	5.4	162	5.2	1.04 (0.84–1.30)

* Rate is given as the number of pneumonia cases per 1,000 person-years. Risk ratio (RR) was estimated using the proportional hazards regression model comparing participants who received vitamin E and those who did not. No covariates were included in the models, because the comparison is between large randomized groups. The sizes of the compared intervention groups are the same within 10% accuracy in the lowest and highest body-weight groups, and within 2% in the other comparisons. The uniformity of the vitamin E effect was tested by adding a dummy variable for vitamin E effect in the 36–59, 60–69, 90–99 and 100–154 kg groups, allowing each of the four groups their own vitamin E supplementation effect. The regression model was improved by χ^2^(4 df) = 7.76, P = 0.10, compared to the model with a uniform vitamin E effect. Adding the vitamin E effect only in the 36–59 and 100–154 kg groups improved the model by χ^2^(2 df) = 6.79, P = 0.034, compared to the model with a uniform vitamin E effect.

† Weight was missing for 12 participants; two of them had pneumonia, one in the vitamin E group and one in the no-vitamin E group.

**Table 3:** Lightest participants (body-weight < 60 kg) initiating smoking at ≤ 20 years: relative risk of hospital-treated pneumonia by vitamin E supplementation

		Intervention		Effect of vitamin E	
		Vitamin E	No vitamin E		
Subgroup	No. of participants	No. of cases	No. of cases	RR (95% CI)*	P-value for interaction
All	935	41	25	1.84 (1.11–3.0)	
BMI †					
< median	467	25	17	1.87 (0.99–3.5)	0.8
≥ median	468	16	8	2.12 (0.90–5.0)	
Height †					
< median	461	16	10	1.91 (0.85–4.3)	0.9
≥ median	474	25	15	1.86 (0.97–3.6)	
Dietary vitamin E †					
< median	467	15	15	1.30 (0.63–2.7)	0.2
≥ median	468	26	10	2.70 (1.30–5.6)	
Dietary vitamin C †					
< median	467	15	16	0.98 (0.48–2.0)	0.026
≥ median	468	26	9	3.48 (1.61–7.5)	
Residual of fruit, vegetables, berries †					
< median	467	19	15	1.53 (0.76–3.1)	0.6
≥ median	468	22	10	2.27 (1.06–4.9)	
β-Carotene supplementation					
No	476	23	12	2.20 (1.06–4.5)	0.7
Yes	459	18	13	1.62 (0.78–3.4)	

* Proportional hazards regression model comparing participants who received vitamin E with those who did not. The regression models were adjusted for age, baseline smoking, intake of coffee and alcohol, BMI and employment. Participants with missing data on confounders (n = 119) are excluded from this table. The sizes of all compared intervention groups are the same within 25% accuracy. RR, risk ratio; CI, confidence interval.

† The medians for the light-weight group are: weight 57.0 kg; BMI 20.0 kg/m^2^; height 168 cm; dietary vitamin E intake 9.1 mg/day; dietary vitamin C intake 75.3 mg/day; residual of fruit, vegetable, and berry intake -2.9 g/day.

**Table 4:** Heaviest participants (body-weight ≥ 100 kg) initiating smoking at ≤ 20 years: relative risk of hospital-treated pneumonia by vitamin E supplementation

		Intervention		Effect of vitamin E	
		Vitamin E	No vitamin E		
Subgroup	No. of participants	No. of cases	No. of cases	RR (95% CI)*	P-value for interaction
All	1226	20	7	3.10 (1.30–7.4)	
BMI †					
< median	613	8	4	2.18 (0.64–7.4)	0.3
≥ median	613	12	3	4.66 (1.30–16.7)	
Height †					
< median	593	11	5	2.19 (0.75–6.3)	0.4
≥ median	633	9	2	5.50 (1.11–27.1)	
Dietary vitamin E †					
< median	613	11	4	3.00 (0.94–9.5)	0.9
≥ median	613	9	3	3.85 (0.93–15.9)	
Dietary vitamin C †					
< median	613	8	6	1.37 (0.46–4.0)	0.019
≥ median	613	12	1	14.5 (1.84–114.5)	
Residual of fruit, vegetables, berries †					
< median	613	12	4	3.55 (1.13–11.2)	0.8
≥ median	613	8	3	2.65 (0.69–10.1)	
β-Carotene supplementation					
No	622	10	3	3.23 (0.89–11.8)	0.9
Yes	604	10	4	3.90 (1.10–13.8)	

* Proportional hazards regression model comparing participants who received vitamin E with those who did not. The regression models were adjusted for age, baseline smoking, intake of coffee and alcohol, BMI and employment. Participants with missing data on confounders (n = 102) are excluded from this table. The sizes of all compared intervention groups are the same within 15% accuracy. RR, risk ratio; CI, confidence interval.

† The medians for the high-weight group are: weight 106.0 kg; BMI 33.5 kg/m^2^; height 179 cm; dietary vitamin E intake 12.4 mg/day; dietary vitamin C intake 95.5 mg/day; residual of fruit, vegetable, and berry intake -4.8 g/day.

In the lowest body-weight participants, the effect of vitamin E supplementation was not modified by BMI, a measure of relative weight (Table 3). In the highest body-weight participants, the harm of vitamin E was more evident in those who had high BMI, but the interaction was not statistically significant (Table 4). Height did not modify the effect of vitamin E supplementation in these subgroups.

Dietary vitamin E intake did not significantly modify the effect of vitamin E supplementation in the low and high weight subgroups. Still, the harm of supplementation was more evident in the low body-weight participants who had high dietary vitamin E intake (Table 3).

Dietary vitamin C intake significantly modified the effect of vitamin E supplementation in both low and high body-weight participants, so that the harm from supplementation was restricted to those with dietary vitamin C intake over the median of the body-weight group (Tables 3 and 4). In the low body-weight group, the interaction between vitamin E supplementation and the dietary vitamin C intake as a continuous variable was significant (P = 0.018), but not in the high body-weight group (P = 0.3).

**Figure 1:**
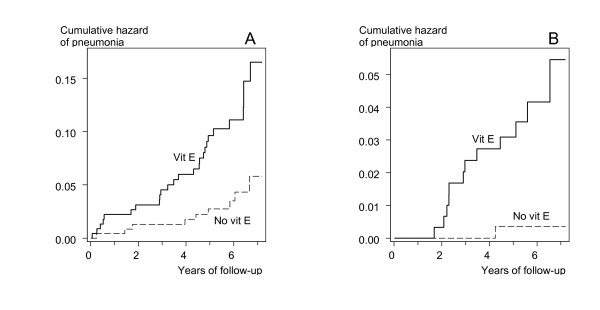
**Vitamin E supplementation and pneumonia risk in subgroups of the ATBC Study participants who started smoking at ≤ 20 years**. A) Weight < 60 kg and dietary vitamin C intake > 75 mg/day (n = 468). B) Weight ≥ 100 kg and dietary vitamin C intake > 95 mg/day (n = 613). Nelson-Aalen cumulative hazard functions for vitamin E and no-vitamin E groups are shown. Each step indicates one case of pneumonia. For the difference between the two survival curves, the logrank test gives A) P = 0.0021 and B) P = 0.0020. The survival curves are cut at 7.2 years because the number of participants declines abruptly thereafter. At 6-years of follow-up there were remaining 114 and 126 participants in A), and 146 and 162 participants in B), in the vitamin E and the no-vitamin E groups, respectively.

Figure 1 shows the survival curves for vitamin E and no-vitamin E participants in the low and high body-weight groups with dietary vitamin C intake over the median. In both groups, the difference between the vitamin E and no-vitamin E groups was highly significant in the logrank test (P = 0.002). In the low body-weight group, the survival curves diverged immediately after the initiation of supplementation (Fig. 1A). However, in the high body-weight group there is a lag period of 1.5 years before cases of pneumonia started to occur in the vitamin E participants (Fig. 1B).

The main food sources for vitamin C are fruit, vegetables and berries and therefore we tested whether some other substances in these foods might explain the effect modification by vitamin C. We modelled fruit, vegetable and berry intake with dietary vitamin C as the explanatory variable, and calculated the residual intake of fruit, vegetables and berries. The residual had no significant association with vitamin E supplementation effect (Tables 3 and 4), indicating that other substances in these foods do not explain the modification by dietary vitamin C.

Level of smoking at baseline and leisure time exercise did not significantly modify the effect of vitamin E supplementation in the low or high body-weight subgroups (data not shown).

In participants with body weight in the middle range from 70 to 89 kg, the effect of vitamin E supplementation was not significantly modified by the baseline variables we tested for the lowest and highest body-weight groups (data not shown).

## Discussion

In this study we surmised that the non-significant increase in pneumonia risk by vitamin E supplementation in the large study cohort might camouflage a greater effect in participants with low body weight because the dose per weight is greater for them. Consistent with our expectation, we found a significant effect by vitamin E supplementation on participants with the lowest body weight, whereas vitamin E had no effect on participants in the middle range of body weight (Table 2). The harm of vitamin E supplementation was restricted to low-weight participants with high dietary vitamin C intake (Table 3, Fig. 1A).

We also observed increased pneumonia risk by vitamin E supplementation in participants with the highest body weight (Table 2). This finding was unexpected and is not consistent with our rationalization for the subgroup analysis by body weight. It is possible that obesity makes people more sensitive to the potential harms of vitamin E. Although our subgroup selection was based on body weight and not BMI, the heaviest-weight participants had a high median BMI of 33.5 kg/m^2 ^and, furthermore, the harm was more pronounced in participants with BMI over the median. In this subgroup, the harm of vitamin E supplementation was also restricted to participants with dietary vitamin C intake above the median (Table 4, Fig. 1B).

In the heavy weight participants with high dietary vitamin C intake, there was a lag period of 1.5 years before cases of pneumonia started to occur in the vitamin E group (Fig. 1B). Vitamin E is a fat soluble antioxidant and it is accumulated in the adipose tissue. In a one-year pharmacokinetic study, high dose α-tocopherol supplementation increased the ratio of α-tocopherol to γ-tocopherol in the adipose tissue continuously over the study [25], and in a three-month study, high dose α-alpha-tocopherol increased buccal mucosa cell vitamin E levels over the study [26], indicating that α-tocopherol accumulation in the adipose tissue is a process of months or years. Accordingly, the lag period in our high weight participants may be explained by slow accumulation of vitamin E in the adipose tissue before the adverse effects start to appear (Fig. 1B).

We assumed that vitamin E supplementation might be more harmful for participants with high dietary vitamin E intake because supplementation could lead to higher systemic levels compared with participants with low dietary vitamin E intakes. In the low body-weight participants we saw a tendency in the expected direction, yet the interaction between dietary and supplemented vitamin E was not statistically significant (Table 3).

Given the proposal that vitamin E supplementation would improve the immune system of elderly people [7,8], our results are alarming. We do not have a biochemical explanation for our findings indicating that vitamin E may increase pneumonia risk in some population groups. Nevertheless, previous reports have indicated that vitamin E may have harmful effects on the immune system [10-14].

In this study, we carried out several subgroup analyses, which might lead to a few low P-values simply because of the multiple comparison problem. However, at both ends of the body-weight scale, the risk of pneumonia was modified by the same variable, namely dietary vitamin C intake, and in both cases the harm was limited to the subgroup with high dietary vitamin C. Furthermore, previously we found that vitamin E supplementation increased the risk of tuberculosis in participants with high dietary vitamin C intake [20], and in this respect the current findings are consistent with the earlier. Furthermore, the P-values testing the divergence between the vitamin E and no-vitamin E groups in the identified subgroups are particularly small, P = 0.002 (Fig. 1), and based on reasonable numbers of cases among several hundreds of participants. On these grounds, the harm observed in the identified subgroups probably is real and not explained by multiple testing.

There is evidence indicating that vitamins E and C interact, and based on biochemical studies, it was proposed that high doses of vitamins C and E might be beneficial for smokers because of the interaction [17]. Furthermore, co-supplementation of high-dose vitamin C and vitamin E is common [1]. We found an effect in the opposite direction so that vitamin E supplementation was harmful when combined with high dietary vitamin C intake (Fig. 1). The vitamin E effect modification by dietary vitamin C was not explained by other substances in fruit, vegetables, and berries. We found a similar harmful interaction between high dietary vitamin C and the effect of vitamin E supplementation on tuberculosis risk [20]. Thus, our findings should lead to caution against a combined high-dose vitamin C and vitamin E supplementation, even though the harm may be restricted to selected groups among smokers.

The current US nutritional recommendations [27] and a recent review [28] conclude that vitamin E is safe at levels up to 1000 mg/day. However, our findings of the ATBC Study indicate that some population groups may be harmed even by a low dose of 50 mg/day (Fig. 1 and refs. [19], [20]). In addition, a Dutch study with elderly people found a significant increase in the severity of respiratory infection by 200 mg/day of vitamin E [29]. Consequently, in some population groups the upper limit of the safe range may be substantially lower than the levels considered conventionally safe.

The two subgroups in which vitamin E increased the risk of pneumonia risk, i.e., the lowest and highest body-weight groups with high vitamin C intake, are small and consist of only 5% of the participant population of the current study (1081/21,657). If these two subgroups are excluded from the study population, the point estimate for the vitamin E supplementation effect on pneumonia risk is reduced from +14% to +4.9%, which demonstrates the restriction of the harm of vitamin E supplementation to these two small subgroups. Nevertheless, the proportion of participants harmed in these two small subgroups, as measured by the increased occurrence of pneumonia, is substantial. In the low body-weight participants with high vitamin C intake, one out of every 13 participants got pneumonia during the trial because of vitamin E supplementation (the number needed to harm, NNH, is 13). In the high body-weight participants with vitamin C intake over the median, the NNH is 28.

There is evidence that vitamin E supplementation may reduce the risk of the common cold and pneumonia in restricted population groups which are so far poorly defined [18,19,30]. However, the observed harms of 50 to 200 mg/day vitamin E supplementation in other population groups indicate that vitamin E can also be detrimental to health. Vitamin E self-supplementation should be discouraged until sub-populations that clearly benefit from the supplementation are characterized properly.

## Abbreviations

ATBC: Alpha-Tocopherol Beta-Carotene Cancer Prevention [Study]; CI: Confidence Interval; FRUVEBE: The total intake of fruit, vegetables and berries; FRUVEBE-RES: The residual in the linear regression model of FRUVEBE as a function of dietary vitamin C intake; RR: Risk Ratio.

## Competing interests

Our study was not funded by sources outside of our university. We have no conflicts of interest.

## Authors' contributions

HH planned the study and wrote the draft of the manuscript and JK participated in planning the analyses and in the critical revision of the manuscript. HH had full access to all of the data in the study and takes responsibility for the integrity of the data and the accuracy of the data analysis.
